# Cerebral perfusion correlates with amyloid deposition in patients with mild cognitive impairment due to Alzheimer's disease

**DOI:** 10.1016/j.tjpad.2024.100031

**Published:** 2025-01-01

**Authors:** Caixia Wang, Deli Ji, Xiao Su, Fang Liu, Yanxin Zhang, Qingzheng Lu, Li Cai, Ying Wang, Wen Qin, Gebeili Xing, Peng Liu, Xin Liu, Meili Liu, Nan Zhang

**Affiliations:** a Department of Neurology, Tianjin Neurological Institute, Tianjin Medical University General Hospital, Tianjin, China, 154 Anshan Road Tianjin 300052, PR China; b Department of Neurology, Baotou Central Hospital, Baotou 014040, PR China; c Department of Neurology, Chifeng Municipal Hospital, Chifeng 024000, PR China; d Inner Mongolia Medical University Affiliated Hospital, Hohhot 010000, PR China; e Department of Neurology, Yulin First Hospital, Yulin City, Shanxi Province 719000, China; f PET/CT Diagnostic Department, Tianjin Medical University General Hospital, Tianjin 300052, PR China; g Department of Radiology, Tianjin Key Lab of Functional Imaging and Tianjin Institute of Radiology, Tianjin Medical University General Hospital, Tianjin 300052, PR China; h Department of Neurology, Inner Mongolia People's Hospital, Hohhot 010000, PR China; i Department of Neurology and Interventional Neurology, The Affiliated Hospital of Qingdao University, No. 16 Jiangsu Road, Qingdao 266000, PR China; j Department of Neurology, Affiliated Hospital of Hebei University, Baoding 071000, PR China; k Department of Neurology, Tianjin Medical University General Hospital Airport Site, Tianjin 300052, PR China

**Keywords:** Mild cognitive impairment, Alzheimer's disease, Cerebral blood flow, Arterial spin labeling, Ad-related perfusion pattern

## Abstract

**Background:**

Changes in cerebral blood flow (CBF) may contribute to the initial stages of the pathophysiological process in patients with Alzheimer's disease (AD). Hypoperfusion has been observed in several brain regions in patients with mild cognitive impairment (MCI). However, the clinical significance of CBF changes in the early stages of AD is currently unclear.

**Objectives:**

The aim of this study was to investigate the characteristics, diagnostic value and cognitive correlation of cerebral perfusion measured with arterial spin labeling (ASL) magnetic resonance imaging (MRI) in patients with MCI due to AD.

**Design, setting and participants:**

A total of fifty-nine MCI patients and 49 cognitively unimpaired controls (CUCs) were recruited and underwent multimodal MRI scans, including pseudocontinuous ASL, and neurocognitive testing. MCI patients were dichotomously classified according to the presence of amyloid deposition on ^11^C-labelled Pittsburgh compound B (PiB) positron emission tomography (PET).

**Measurements:**

The differences in CBF and expression of the AD-related perfusion pattern (ADRP), established by spatial covariance analysis in our previous study, were compared between the PiB+ MCI group and the CUC group and between the PiB+ and PiB- MCI groups. The diagnostic accuracy and correlations with cognitive function scores for CBF and ADRP expression were further analyzed.

**Results:**

Hypoperfusion in the precuneus and posterior cingulate cortex (PCC) was more characteristic of patients with MCI due to AD than of those with non-AD-related MCI. The relative regional CBF value of the left precuneus best distinguished patients with MCI due to AD from CUCs and patients with MCI due to non-AD conditions. Cerebral perfusion, as indicated by either the relative regional CBF or the expression score of the ADRP, was strongly correlated with certain cognitive function scores.

**Conclusions:**

Here, we show that changes in CBF in the precuneus/PCC are promising MRI biomarkers for the identification of an AD etiology in patients with MCI. ASL, a noninvasive and cost-effective tool, has broad application prospects in the screening and early diagnosis of AD.

## 1 Introduction

As the population of elderly individuals continues to grow, dementia, especially Alzheimer's disease (AD), will impose greater mental, psychological and socioeconomic burdens on global public health [1]. The neuropathological hallmarks of AD include extracellular amyloid plaques and intracellular neurofibrillary tangles principally composed of hyperphosphorylated tau, which can be detected early or reflected by positron emission tomography (PET) and cerebrospinal fluid (CSF) as disease biomarkers according to recent diagnostic criteria [2,3]. However, the high cost and associated radiation exposure of PET and the invasiveness of lumbar puncture for CSF collection impede the clinical application of these two techniques. An alternative biomarker for screening and evaluating the early stages of AD that could be easily and safely obtained is still desirable, particularly in the era of disease-modifying therapy.

In addition to amyloid-β (Aβ) and tau, other pathophysiological processes undoubtedly participate in the onset and progression of AD. Recent studies have reported a strong correlation between vascular dysfunction and the pathogenesis of AD [4]. Vascular abnormalities can be observed in the AD brain during the early pathological stages that progressively worsen throughout the course of the disease [5]. Relatedly, a hypothetical model of AD biomarkers suggests that vascular dysfunction, such as changes in the cerebral blood flow (CBF) and blood–brain barrier, may contribute to the initial stages of the pathophysiological process in patients with AD, even before the emergence of Aβ and tau pathologies [6]. Therefore, methods for assessing CBF would be a promising tool for early diagnosis and disease monitoring in patients diagnosed with the spectrum of AD [7].

Arterial spin labeling (ASL) is a magnetic resonance imaging (MRI) technique that measures tissue perfusion according to the CBF and can be used to reflect neurodegenerative processes and neuronal activities [[8], [9], [10], [11]]. Compared with other perfusion measurements, such as single photon emission computed tomography (SPECT) and PET, ASL has many advantages, such as greater safety, noninvasiveness, a lack of radiation exposure (as arterial blood water is used as an endogenous tracer), good reproducibility, and ease of operability [8,12]. We previously demonstrated global and regional CBF changes as measured with ASL in patients with mild to moderate AD and further established an AD-related perfusion pattern (ADRP) via multivariate spatial covariance analysis of a scaled subprofile model (SSM) based on principal component analysis (PCA). The ADRP features negative loading in the bilateral middle and posterior cingulate and precuneus, inferior parietal lobule, and frontal areas and positive loading in the cerebellum and deep gray matter (GM), and in an assessment of AD patients, it showed good diagnostic performance and strong correlations with cognitive function [13]. However, the expression of ADRP in the early stages of AD is unknown.

The pathophysiological changes in AD occur approximately 20 years before symptoms develop. Mild cognitive impairment (MCI), as the prodromal and earliest stage of AD according to clinical observations, deserves particular attention [14]. CBF changes are related to cognitive function and have predictive value for disease progression in MCI patients [[15], [16], [17]]. Hypoperfusion has been observed in several brain regions in various studies, including the frontal, temporal, parietal, and occipital lobes, posterior cingulate cortex (PCC), precuneus, and hippocampus, in MCI patients [[18], [19], [20], [21], [22], [23]], while hyperperfusion has also been reported in a few brain areas, such as the hippocampus, amygdala, and deep GM [20]. However, many of these studies were obtained from MCI patients diagnosed clinically but with an unclear amyloid status. Therefore, the perfusion changes in patients with MCI due to AD should be further clarified, and their diagnostic value and suitability as biomarkers in the early stages of AD continuum should be determined.

In this study, we assessed changes in both the CBF value and perfusion pattern expression in patients with MCI due to AD (supported by amyloid PET) with respect to cognitively unimpaired controls (CUCs) and assessed their diagnostic value. Moreover, the differential value of perfusion outcomes was investigated between patients with MCI due to AD and those with non-AD-related MCI. Furthermore, the correlations between cognitive function with various domains and cerebral perfusion were analyzed for patients with MCI due to AD or those with MCI.

## 2 Methods

### 2.1 Participants

This study was approved by the Ethics Committee of Tianjin Medical University General Hospital. Fifty-nine patients with MCI and 49 age- and sex-matched CUCs were consecutively recruited from the memory clinic of the hospital. Written informed consent was obtained from all participants or their legally designated representatives prior to inclusion. All participants were aged 50–85 years and underwent a standard clinical evaluation, including demographic and medical history, physical and neurological examinations, comprehensive neuropsychological assessments, laboratory tests, and brain MRI. No participant was found to have moderate or severe stenosis in the large vessels according to cranial magnetic resonance angiography (MRA), carotid duplex ultrasound, or transcranial Doppler (TCD).

Patients with MCI were those who met the criteria for mild neurocognitive disorder according to the fifth edition of the Diagnostic and Statistical Manual of Mental Disorders (DSM-5) [24] and the recommendations of Petersen's criteria for the amnestic type of MCI [25], including (1) subjective memory complaints, corroborated by an witness; (2) objective memory impairment indicated by 1.0 or more standard deviations (SDs) below the age- and education-corrected norm for delayed recall on the Logical Memory subtest of the Wechsler Memory Scale-Chinese, Revised [26]; and (3) preserved general cognitive function and activities of daily living (ADL) and a lack of dementia, with a Mini-Mental State Examination (MMSE) score≥24 and a Clinical Dementia Rating (CDR) score=0.5. Patients whose cognitive impairment was induced by specific causes, such as frontotemporal dementia (FTD), dementia with Lewy bodies (DLB), hydrocephalus, cerebrovascular diseases, multiple sclerosis, alcohol or drug abuse, severe depression, thyroid dysfunction, vitamin B12 deficiency, human immunodeficiency virus infection, or neurosyphilis, were excluded.

The inclusion criteria for CUCs included the following: (1) no subjective cognitive decline complaints and normal performance in each cognitive domain of objective neuropsychological tests, as mentioned in the section of neuropsychological assessments below; (2) a CDR score=0, MMSE score > 26, 17 item-Hamilton Depression Scale (HAMD) score < 17; and (3) no clinically significant brain atrophy (medial temporal lobe atrophy score=0–1 for subjects < 75 years old or 0–2 for subjects ≥75 years old) or cerebrovascular lesions (Fazekas score < 2) [27] on brain MRI. Individuals with a definite family history of familial AD or other types of dementia, a history of neurological or mental disorders, such as cerebrovascular diseases, epilepsy, alcohol or drug abuse, or depression, were excluded.

### 2.2 Neuropsychological assessment

All participants received a comprehensive neuropsychological assessment, as described in our previous publications [13,28], including the Rey Auditory Verbal Learning Test (AVLT), the Brief Visuospatial Memory Test Revised (BVMT-R), the Symbol Digit Modalities Test (SDMT), the Trail Making Test-A (TMT-A) and TMT-B, the Stroop Color-Word Test (SCWT), the Verbal Fluency Test (VFT), the Controlled Oral Word Association Test (COWAT), the Boston Naming Test (BNT), and the Benton Judgment of Line Orientation (JLO). Raw scores were converted to z scores using the means and SDs of the scores of all the CUCs included in this study. The z scores of the TMT‐A and the TMT‐B were multiplied by −1 to be consistent with the scores of the other tests, in which higher scores indicate better performance. The scores for five main cognitive domains were calculated: (1) memory (total)=average of the scores for the total learning, delayed recall and recognition domains on the AVLT and the BVMT-R; and memory (recall)=average of the delayed recall score of the AVLT and the BVMT-R; (2) attention and information processing speed=average of the scores of the SDMT, the TMT-A, and word reading and color naming domains of the SCWT (SCWT‐W and ‐C); (3) executive function=average scores of the TMT-B and color‐word condition domain of the SCWT (SCWT-CW); (4) language=average of the scores of the VFT, the COWAT and the BNT; and (5) visuospatial function=the JLO score.

### 2.3 PET imaging and interpretation

All MCI patients underwent ^11^C-labeled Pittsburg compound B (PiB) PET imaging at the PET/CT center of Tianjin Medical University General Hospital on a Discovery PET/CT 710 scanner (GE Healthcare) in three-dimensional scanning mode. A bolus of PiB was injected into the antecubital vein at a mean dose of 370–555 MBq. Images were acquired during a 90-min dynamic PET scan (34 frames: 4 × 15 s, 8 × 30 s, 9 × 60 s, 2 × 180 s, 8 × 300 s, 3 × 600 s). Four cortical areas on each side, including the frontal lobe, lateral temporal lobe, lateral parietal lobe, precuneus/posterior cingulate gyrus, and occipital lobe, were evaluated for PiB uptake. The image was considered as amyloid PET positive when one target region with increased cortical PiB uptake was observed. This visual rating procedure was conducted by two experienced nuclear medicine physicians for a consensus, and has been validated to be consistent with the quantitative analysis described in our previous studies [29,30]. Patients were then dichotomously classified into a PiB-positive (+) group and a PiB-negative (-) group.

### 2.4 MRI acquisition

The imaging acquisition was performed for all participants on a 3.0-Tesla MRI scanner (Discovery MR750, General Electric, Milwaukee, WI, USA) with a 64-channel phased array head coil. The coronal T1-weighted 3D brain volume sequence was first acquired to serve as a template for coregistration with ASL imaging data with the following parameters: echo time/repetition time (TE/TR): 3.2 ms/8.2 ms; flip angle (FA): 12°; field of view (FOV): 256×256×188 mm^3^; matrix size: 256×256; NEX=1; slice thickness: 1.0 mm; and number of slices: 188. The 3D pseudocontinuous ASL (PCASL) series was prepared to measure whole-brain perfusion via 3D fast spin‒echo acquisition and background suppression with the following parameters: TE/TR: 11.1 ms/5046 ms; labeling duration: 1450 ms; postlabeling delay (PLD): 2025 ms; FA: 111°; matrix size: 128×128; FOV: 240×240×150 mm^3^; arms=8; acquisition points=512; slice thickness: 3 mm; and number of slices: 50. A proton density image was also acquired at the same time to quantify the CBF from the ASL series. During the resting-state ASL scan, the participants had their ears plugged and were instructed to keep their eyes closed, not to think of anything in particular, and not to fall asleep.

### 2.5 MRI data preprocessing

Statistical parametric mapping (SPM12, Institute of Neurology, London, UK) running in MATLAB (Version R2018b; MathWorks, Natick, MA, USA) on a Windows computer was used to preprocess all the images of the participants. All the images were manually checked by a trained investigator to assess image quality and ensure successful coregistration. The DICOM-format images were converted to NIFTI format with MRIcron. Image preprocessing was conducted as follows: (1) CBF images were registered to the structural MR images (linear deformation, 4th-degree B-spline); (2) the structural images were normalized to the standard Montreal Neurological Institute (MNI) brain template and segmented into GM, white matter (WM), and CSF probability maps; (3) the CBF images were normalized using the parameters determined from the structural images and multiplied by a binary brain tissue mask consisting of only GM and WM; (4) normalized CBF maps were then smoothed by using a with a 10-mm full-width at half-maximum (FWHM) Gaussian kernel. All the processed images of the CBF and tissue maps had matrix dimensions of 121×145×121 and a voxel size of 1.5 × 1.5 × 1.5 mm^3^ in the MNI space.

### 2.6 Brain mapping analysis with SPM

Univariate analysis was performed with SPM12 software. CBF differences between the PiB+ MCI and CUC groups and between the PiB+ and PiB- MCI groups were assessed with the two-sample *t-*test model, with age and sex used as covariates. With a voxel-level peak threshold of *P* < 0.001 over whole brain regions, we selected all clusters of voxels without/with adjustment for global values with analysis of covariance (ANCOVA). The coordinates of the resulting topography were reported in the standard MNI anatomical space. The anatomical locations and corresponding Brodmann areas (BAs) were obtained via MRIcron software. The nearest GM locations were reported for all these regions. Areas of altered (decreased or increased) perfusion were overlaid on a standard T1-weighted MRI brain template in stereotaxic space with MRIcro software.

To quantify CBF changes in specific cortical regions, we used a 4-mm radius spherical volume of interest (VOI) centered at the peak voxels of clusters that were significantly different in the SPM analyses between the PiB+ MCI group and the CUC group and between the PiB+ MCI group and the PiB- MCI group. We then obtained the relative CBF values by calculating the ratio of the specific regional CBF values to the global CBF values in all participants with SPM12.

### 2.7 Subject expression of the ADRP

The ADRP was established in our previous study [13]. In this study, a subject expression score was obtained for each CBF image on a prospective single-case basis via a voxel-based algorithm in the SSM/PCA toolbox (available at http://www.feinsteinneuroscience.org) [31]. The corresponding subject expression scores reflect the degree to which each subject expresses this pattern.

### 2.8 Statistical analysis

The statistical analysis of the vector data was performed with SPSS 26.0 (SPSS, Inc., Chicago, IL, USA), unless specified otherwise, by one investigator who was unaware of the clinical groups and related information. All the tests were two-tailed, and values of *P* < 0.05 were considered to indicate statistical significance. The demographic and clinical data of all participants were analyzed with the Pearson chi‐square test for categorical variables or two-sample *t*-tests (between the MCI group and the CUC group) and one‐way analysis of variance (ANOVA) followed by the Bonferroni correction for post hoc pairwise comparisons (between the PiB+ MCI group, the PiB- MCI group, and the CUC group) for continuous variables. The differences in global CBF values, relative regional CBF values, and subject ADRP expression scores were compared between the PiB+ MCI group and the CUC group and between the PiB+ MCI group and the PiB- MCI group separately with two-sample *t*-tests. Receiver operating characteristic (ROC) curve analysis was then performed to assess the performance of the CBF values and subject ADRP expression as predictors of disease status with GraphPad Prism version 8.3 for Windows (GraphPad Software, San Diego, CA, USA). Pearson correlation analysis was performed to assess the correlations between global CBF values, relative regional CBF values, and subject ADRP expression scores and cognitive function scores in each domain in both patients with MCI due to AD and all MCI patients, followed by the Bonferroni correction for multiple comparisons.

## 3 Results

### 3.1 Demographic and clinical profiles of the study population

The demographic and clinical characteristics of the participants are summarized in Table 1. No significant differences in age, education, handedness, and vascular risk factors, including hypertension, hyperlipidemia, obesity and smoking, were observed between the PiB+ MCI group, the PiB- MCI group, and the CUC group (Table 1) or between the MCI group and the CUC group (Supplementary Table 1). Although MCI patients included a higher proportion of females compared with CUCs (Supplementary Table 1), no significant difference in the sex distribution was observed between the PiB+ MCI group, the PiB- MCI group, and the CUC group (Table 1). Both the PiB+ MCI and PiB- MCI groups had lower scores on the MMSE and memory domain subtest than the CUC group did (*P* < 0.05). Compared with the CUC group, the PiB+ MCI group also presented with lower scores in the processing speed, executive function, language and visuospatial function domains (*P* < 0.05). Although there was no significant difference in the MMSE score between the two MCI groups, the PiB+ MCI group presented with lower scores in the memory, processing speed and executive function domains than the PiB- MCI group did (*P* < 0.05).

**Table 1:** Demographics and cognitive performance of all participants.

	PiB+ MCI *N* = 41	PiB- MCI *N* = 18	CUC *N* = 49	F/χ2	P
Age, years	69.39 (5.87)	67.50 (5.27)	67.45 (5.51)	*F*=1.50	0.23
Sex, F/M	32/9	15/3	29/20	χ2=5.62	0.06
Education, years	12.34 (3.11)	11.83 (2.94)	11.82 (3.53)	*F*=0.32	0.73
Handedness, L/R	1/40	2/16	1/48	χ2=2.49	0.29
Hypertension	11/30	9/9	21/28	χ2=3.83	0.15
Hyperlipidemia	7/34	5/13	9/40	χ2=0.92	0.63
Obesity	1/40	1/17	5/44	χ2=2.42	0.30
Smoking	2/39	0/18	2/47	χ2=1.52	0.47
MMSE^a^^,^^b^	25.20 (1.52)	25.94 (1.39)	27.71 (1.63)	*F*=30.56	<0.01
Memory (total)^a^^,^^b^^,^^c^	−2.27 (1.05)	−0.90 (1.33)	0.00 (0.68)	*F*=62.89	<0.01
Memory (recall)^a^^,^^b^^,^^c^	−2.43 (1.17)	−1.07 (1.42)	0.00 (0.68)	*F*=62.35	<0.01
Processing speed^a^^,^^c^	−1.14 (1.02)	−0.24 (0.75)	0.00 (0.74)	*F*=20.43	<0.01
Executive function^a^^,^^c^	−1.46 (1.20)	−0.19 (1.06)	0.00 (0.82)	*F*=24.56	<0.01
Language^a^	−0.26 (1.80)	−0.47 (0.73)	0.00 (0.58)	*F*=12.08	<0.01
Visuospatial function^a^	−0.82 (1.63)	−0.24 (1.60)	0.00 (1.00)	*F*=4.02	0.02

The data are presented as the means (SDs) unless otherwise specified. Z scores for different cognitive domains were calculated from the raw scores of neuropsychological assessments according to the mean and SDs of the scores of all CUCs.

PiB, ^11^C-Pittsburgh compound B; MCI, mild cognitive impairment; CUC, cognitively unimpaired control; MMSE, Mini-Mental State Examination.

a Comparison between PiB+ and CUC, *P* < 0.05.

b Comparison between PiB- and CUC, *P* < 0.05.

c Comparison between PiB+ and PiB-, *P* < 0.05.

### 3.2 Voxel-based CBF changes from univariate analysis

Before adjusting for the global value, relative to the CUC group, the PiB+ MCI group presented with decreased absolute CBF in the left fusiform gyrus, left inferior parietal lobule, left middle cingulate cortex (MCC), and right angular gyrus, but no areas presented with increased CBF (Supplementary Table 2, Supplementary Fig. 1).

**Fig. 1:**
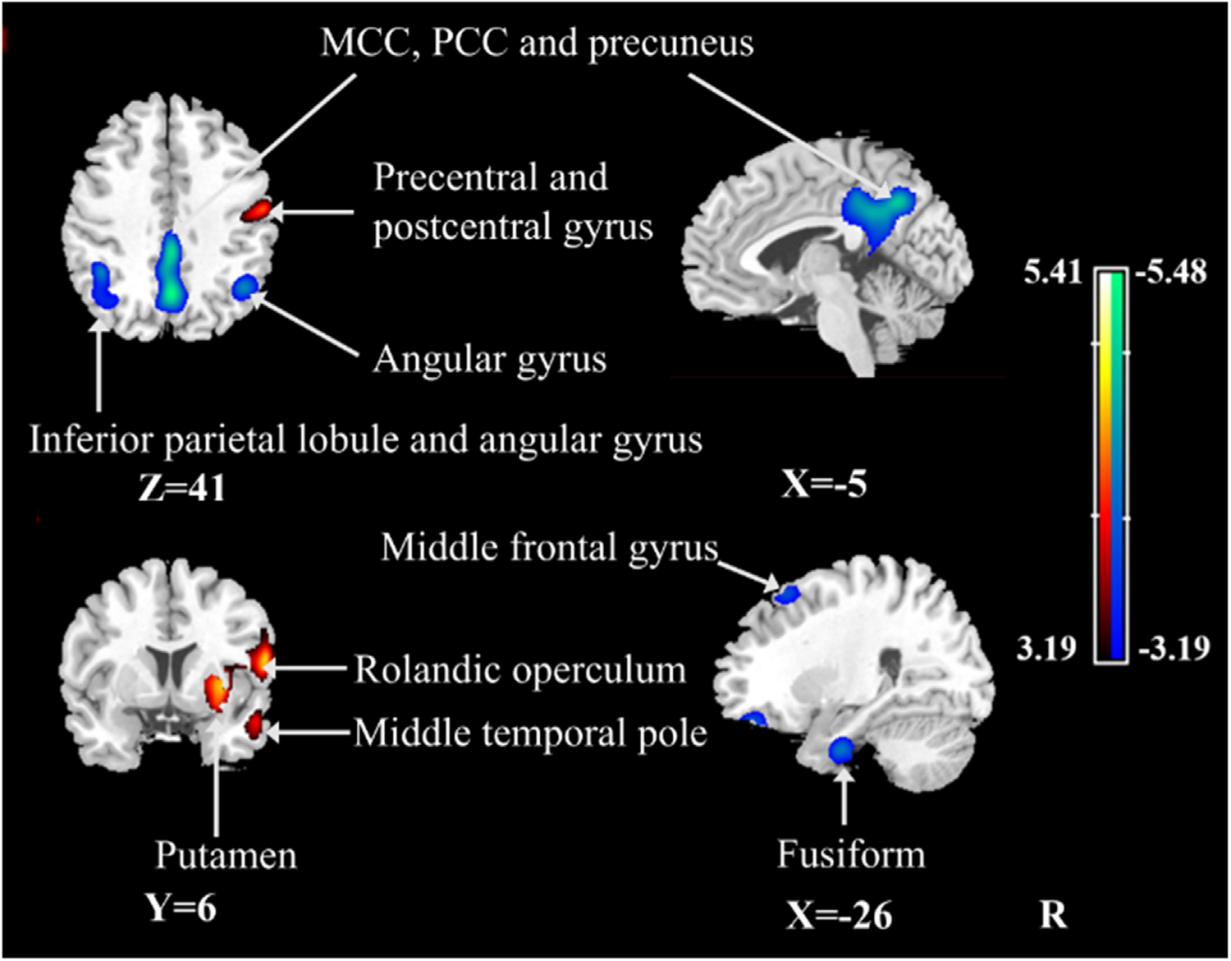
Regional changes in relative CBF after ANCOVA normalization of the global value in patients with MCI due to AD compared to CUCs. Cooler colors indicate regions with decreased relative CBF, and warmer colors indicate regions with increased relative CBF in patients with MCI due to AD with respect to CUCs. A threshold of 3.19 (*P* < 0.001, uncorrected) was used to overlay the SPM maps onto a standard MRI brain template. CBF, cerebral blood flow; ANCOVA, analysis of covariance; MCI, mild cognitive impairment; AD, Alzheimer's disease; CUC, cognitively unimpaired control; SPM, statistical parametric mapping; MRI, magnetic resonance imaging; MCC, middle cingulate cortex; PCC, posterior cingulate cortex.

After ANCOVA adjustment for the global value, the PiB+ MCI group demonstrated both decreased and increased CBF in different regions with respect to the CUC group (Table 2, Fig. 1). Specifically, regions with relative hypoperfusion included the left middle frontal gyrus, bilateral angular gyrus, left fusiform gyrus, left MCC, PCC and bilateral precuneus, whereas regions with relative hyperperfusion included the right precentral gyrus, right postcentral gyrus, right middle temporal pole, right Rolandic operculum, right rectus, right putamen, and right caudate.

**Table 2:** Regions showing CBF changes in patients with MCI due to AD compared with CUCs (ANCOVA normalization).

Structure	BA	X	Y	Z	T	Cluster size (ml)
Decreased CBF						
Left precuneus a	7	0	−60	41	5.48	14.48
Left middle cingulate cortex a	23	−2	−45	35	5.46	b
Right precuneus	7	5	−42	8	3.30	b
Left inferior parietal lobule a	40	−44	−48	50	5.38	6.22
Left angular gyrus	39	−36	−63	45	3.57	b
Right angular gyrus	39	42	−57	41	4.41	3.69
Left fusiform	37	−26	−9	−36	4.28	2.56
Left middle frontal gyrus	9	−26	24	57	3.91	1.03
Increased CBF						
Right putamen a	48	32	−2	−5	5.41	20.60
Right postcentral a	43	60	−11	24	5.39	b
Right Rolandic operculum a	6	56	6	15	4.69	b
Right caudate	–	21	20	8	3.98	b
Right rectus	11	15	20	−14	3.56	b
Right precentral	4	42	−17	53	3.57	b
Right middle temporal pole	21	51	8	−20	3.98	1.39

Significant clusters were defined as those for which the uncorrected *P* < 0.001.

a Regions that survived the FWE correction at *P* < 0.05.

b Regions belong to the same cluster for which the volume is provided above. CBF, cerebral blood flow; MCI, mild cognitive impairment; AD, Alzheimer's disease; CUC, cognitively unimpaired control; ANCOVA, analysis of covariance; BA, Brodmann area; FWE, family wise error.

**Fig. 2:**
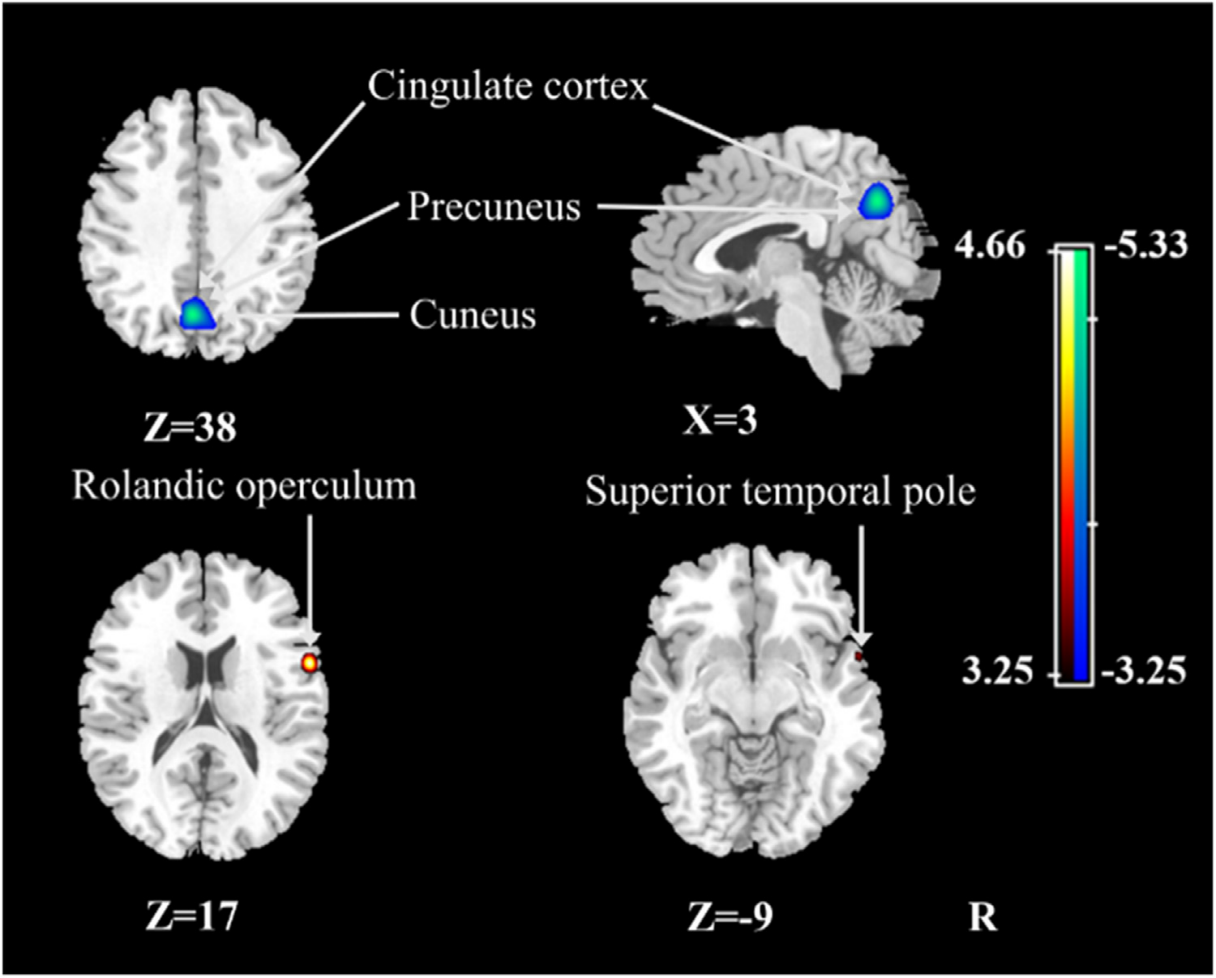
Regional changes in relative CBF after ANCOVA normalization of the global value in patients with MCI due to AD compared with patients with MCI due to non-AD conditions. Cooler colors indicate regions with decreased relative CBF, and warmer colors indicate regions with increased relative CBF in patients with MCI due to AD with respect to patients with MCI due to non-AD conditions. A threshold of 3.25 (*P* < 0.001, uncorrected) was used to overlay the SPM maps onto a standard MRI brain template. CBF, cerebral blood flow; ANCOVA, analysis of covariance; MCI, mild cognitive impairment; AD, Alzheimer's disease; SPM, statistical parametric mapping; MRI, magnetic resonance imaging.

Without adjusting for the global value, the PiB+ group showed neither decreased nor increased CBF in any brain area relative to the PiB- group. After ANCOVA adjustment for the global value, the PiB+ MCI group demonstrated both increases and decreased in CBF in different regions with respect to the PiB- MCI group (Table 3, Fig. 2). Specifically, compared with the PiB- MCI group, the PiB+ MCI group presented decreased CBF in the right cingulate gyrus, bilateral precuneus and right cuneus and increased CBF in the right Rolandic operculum and right superior temporal pole.

**Table 3:** Regions showing CBF changes in patients with MCI due to AD compared with patients with MCI due to non-AD conditions (ANCOVA normalization).

Structure	BA	X	Y	Z	T	Cluster size (ml)
Decreased CBF						
Left precuneus^a^	7	−5	−65	38	5.33	3.75
Increased CBF						
Right Rolandic operculum	6	56	8	17	4.66	1.76
Right superior temporal pole	38	57	8	−9	3.48	0.13

Significant clusters were defined as those for which the uncorrected *P* < 0.001.

a Regions that survived FWE correction at *P* < 0.05. CBF, cerebral blood flow; MCI, mild cognitive impairment; AD, Alzheimer's disease; ANCOVA, analysis of covariance; BA, Brodmann area; FWE, family wise error.

### 3.3 Discriminative value of global CBF, regional CBF, and ADRP expression for MCI

**Fig. 3:**
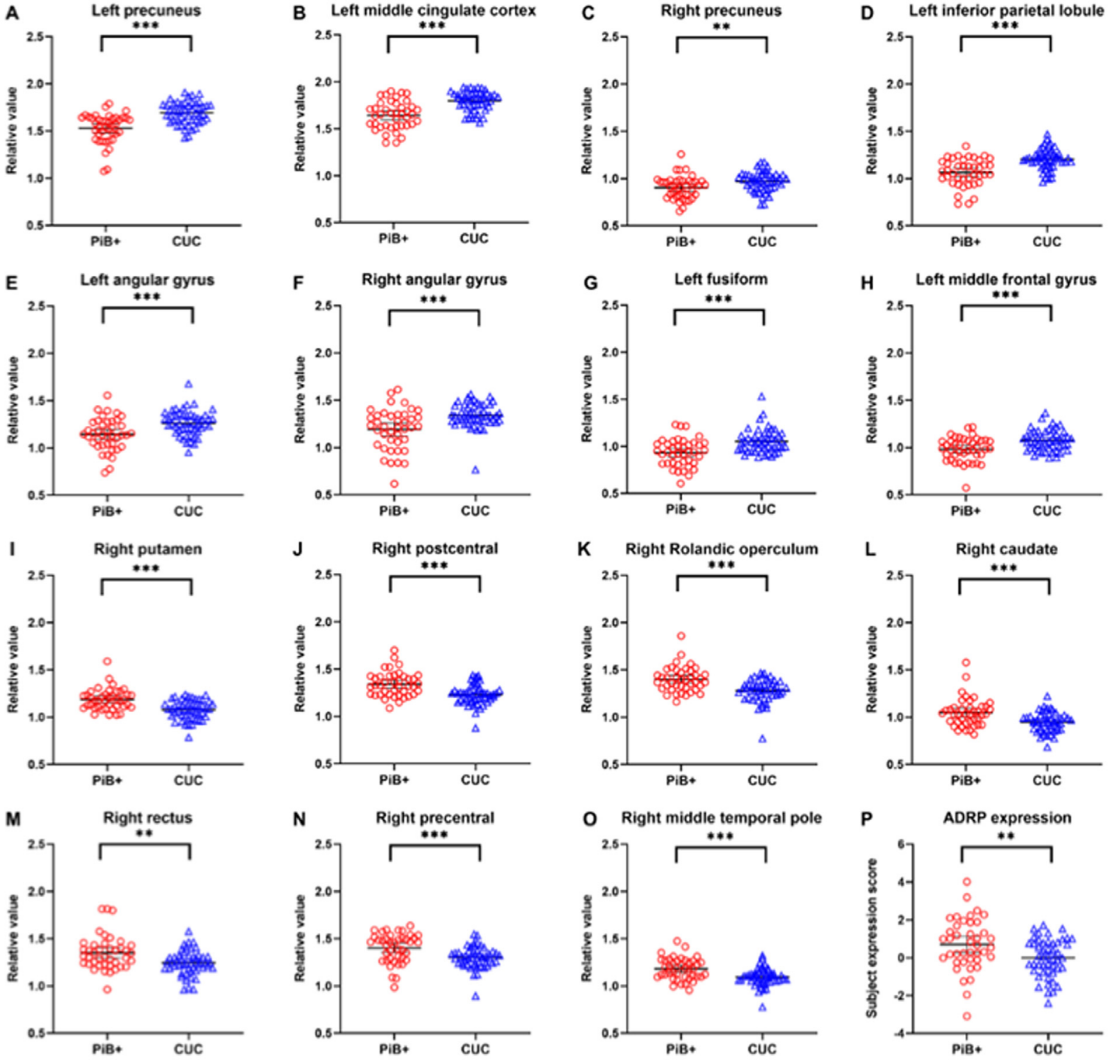
Differences in the relative CBF values for fifteen sample regions and the subject expression scores of the ADRP between the PiB+ MCI group and the CUC group. (A–O) Comparisons of relative CBF values in the left precuneus (0, −60, 41), left middle cingulate cortex (−2, −45, 35), right precuneus (5, −42, 8), left inferior parietal lobule (−44, −48, 50), left angular gyrus (−36, −63, 45), right angular gyrus (42, −57, 41), left fusiform (−26, −9, −36), left middle frontal gyrus (−26, 24, 57), right putamen (32, −2, −5), right postcentral gyrus (60, −11, 24), right Rolandic operculum (56, 6, 15), right caudate (21, 20, 8), right rectus (15, 20, −14), and right precentral gyrus (42, −17, 53), right middle temporal pole (51, 8, −20) between patients with MCI due to AD and CUCs, obtained post hoc within a spherical volume of interest (4 mm radius). (P) Comparison of ADRP expression between patients with MCI due to AD and CUCs. CBF, cerebral blood flow; ADRP, AD-related perfusion pattern; PiB, ^11^C-Pittsburgh compound B; MCI, mild cognitive impairment; CUC, cognitively unimpaired control. **P < 0.01; ***P < 0.001.

Between the PiB+ MCI group and the CUC group, there was no significant difference in the global value measured from the CBF map (36.94±5.74 vs. 37.71±5.15 ml/100 g/min, *t*=−0.673, *P*=0.503); sample plots of the relative CBF values for the major regions are shown in Fig. 3A-O. The ADRP expression score was significantly greater in PiB+ MCI patients than in CUCs (0.70±1.37 vs. 0.00±1.00, *t*=2.800, *P*=0.006; Fig. 3P). In terms of relative regional CBF values, those of the left precuneus demonstrated the highest area under the ROC curve (AUC=0.80, 95 % confidence interval 0.71–0.89) in distinguishing PiB+ MCI patients from CUCs, with a sensitivity of 61.22 % and a specificity of 87.80 %, followed by the relative CBF values of the left MCC, left inferior parietal gyrus, right putamen, and right Rolandic operculum, all of which yielded AUCs > 0.75 (Supplementary Fig. 2). The subject expression score of the ADRP had an AUC of 0.67 (95 % confidence interval 0.55–0.78), with a sensitivity of 46.30 % and a specificity of 83.70 %, in differentiating PiB+ MCI patients from CUCs.

**Fig. 4:**
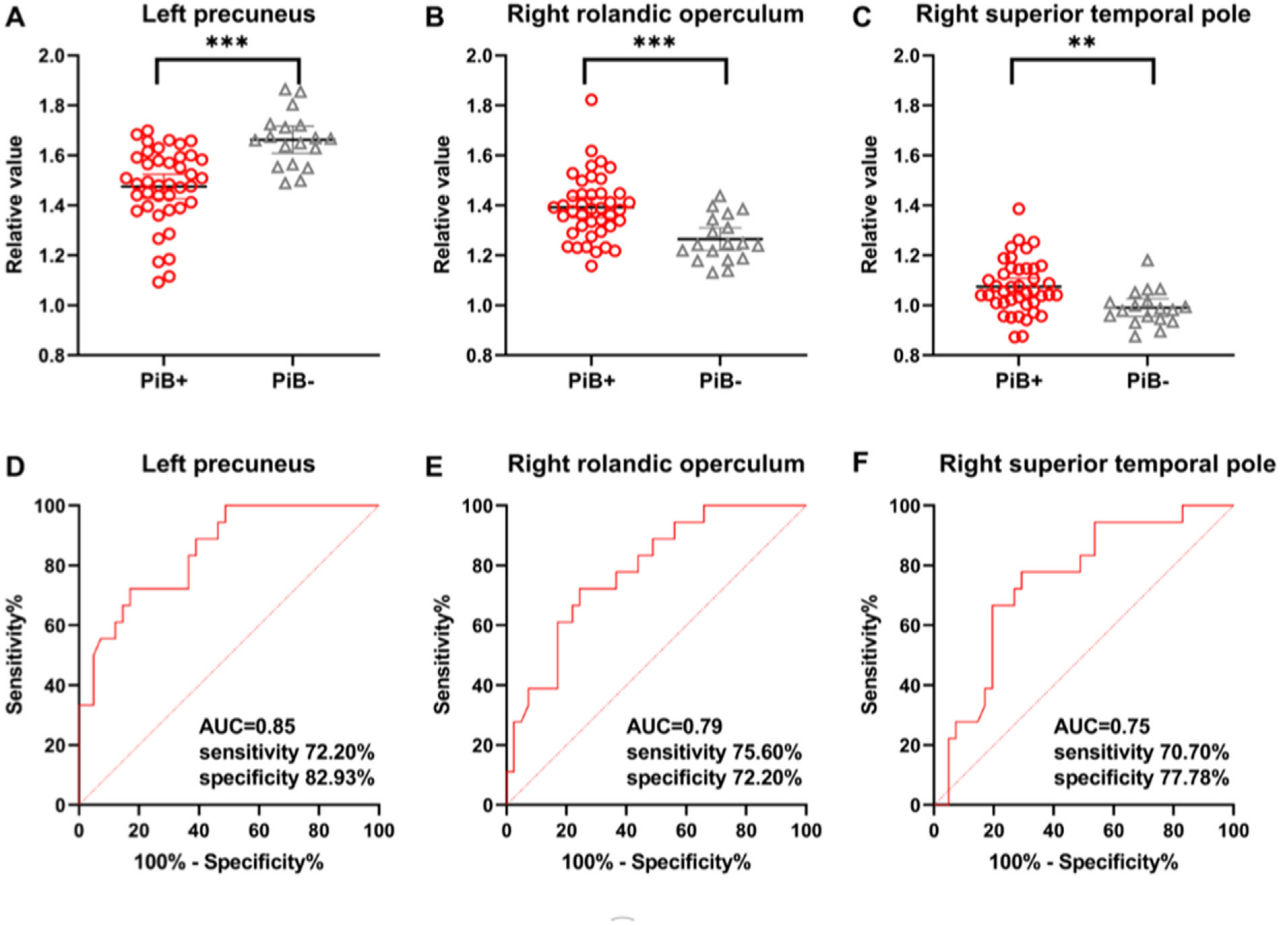
Differences and ROC curves of the relative CBF values between the PiB+ MCI group and the PiB- MCI group. (A-C) Comparisons of relative CBF values in the left precuneus (−5, −65, 38), right Rolandic operculum (56, 8, 17), and right superior temporal pole (57, 8, −9) between the PiB+ MCI group and the PiB- MCI group, obtained post hoc within a spherical volume of interest (4 mm radius). (D-F) ROC curves of relative CBF values in discriminating between patients with MCI due to AD and those with MCI due to non-AD conditions. ROC, receiver operating characteristic; CBF, cerebral blood flow; PiB, ^11^C-Pittsburgh compound B; MCI, mild cognitive impairment; AD, Alzheimer's disease; AUC, area under the curve. **P < 0.01; ***P < 0.001.

Between the PiB+ MCI group and the PiB- MCI group, there was no significant difference in the global CBF value (36.94±5.74 vs. 37.48±7.41 ml/100 g/min, *t*=−0.304, *P*=0.762). Compared with the PiB- MCI group, the PiB+ MCI group demonstrated lower relative CBF values in the left precuneus and greater relative CBF values in the right Rolandic operculum and right superior temporal pole (Fig. 4A-C). The ADRP expression scores (0.70±1.37 vs. 0.12±1.43, *t*=1.478, *P*=0.145) did not differ significantly between the PiB+ MCI group and the PiB- MCI group. ROC curve analyses of these values for distinguishing MCI patients with PiB+ from those with PiB- (Fig. 4D-F) revealed that the relative regional CBF value of the left precuneus had the maximum AUC of 0.85 (95 % confidence interval 0.75–0.95), as well as a sensitivity of 72.20 % and a specificity of 82.93 %.

### 3.4 Cognitive correlations of perfusion in PiB+ MCI patients and all MCI patients

**Fig. 5:**
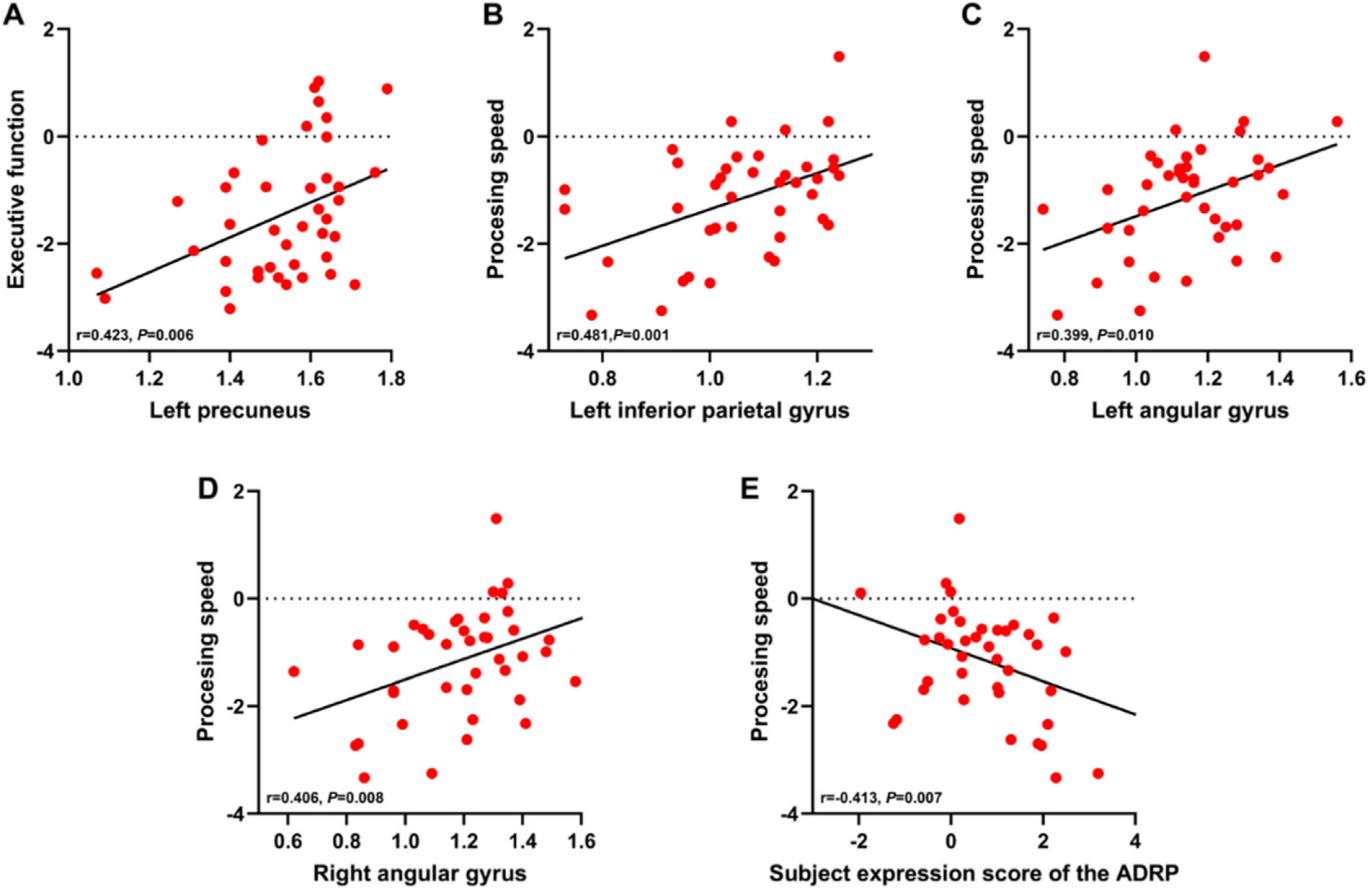
Correlations between relative regional CBF, the subject expression score of the ADRP and cognitive function scores in patients with MCI due to AD. Raw scores were converted to z scores for the subject expression score of the ADRP and the scores of the different cognitive domains. Pearson correlation analysis was used with subsequent application of the Bonferroni correction for multiple comparisons. CBF, cerebral blood flow; ADRP, AD-related perfusion pattern; MCI, mild cognitive impairment; AD, Alzheimer's disease.

The correlations between the cognitive function scores in various domains and global and regional (relative values of regions showing differences in the specific group comparisons) CBF and ADRP expression scores in both patients with MCI due to AD and all MCI patients were analyzed. In PiB+ MCI patients, the relative CBF value of the left precuneus was positively correlated with executive function, and the left inferior parietal lobule and bilateral angular gyrus relative CBF values were positively correlated with processing speed after Bonferroni correction (Fig. 5A-D). There was a negative correlation between the subject ADRP expression score and processing speed (Fig. 5E), but no significant associations were observed between the global CBF and any cognitive function score.

In all MCI participants, the relative CBF values of the left precuneus, left inferior parietal lobule, and left angular gyrus were positively correlated with processing speed and executive function scores; the left MCC was positively correlated with the memory score; the right angular gyrus was positively correlated with memory, processing speed and executive function scores; the right postcentral gyrus was negatively correlated with the processing speed score; the Rolandic operculum was negatively correlated with processing speed and executive function scores; and the right caudate was negatively correlated with executive function score (Supplementary Fig. 3A-N). There was a negative correlation between the subject ADRP expression score and processing speed and executive function scores (Supplementary Fig. 3O-P), but no significant associations were observed between the global CBF and any cognitive function score.

## 4 Discussion

In this study, regional CBF changes were identified in patients with MCI due to AD and compared with those in MCI patients without amyloid deposition on PET. The relative CBF value of the left precuneus performed best in both distinguishing patients with MCI due to AD from CUCs or in differentiating patients with MCI due to AD from patients with MCI due to non-AD conditions. Cerebral perfusion, as indicated by either regional CBF or the ADRP expression score, was correlated with a variety of cognitive function scores.

We found that patients with MCI due to AD had brain regions demonstrating hypoperfusion, including the inferior parietal lobe, precuneus, MCC and PCC. Previous findings have been inconsistent to a certain degree [[18], [19], [20], [21], [22]]. Several factors may have influenced these discrepancies across studies, such as the sample size, diagnostic criteria, phenotype, and the imaging modalities and therapeutic interventions employed [32,33], in particular the heterogeneous etiology, as CBF changes could be derived from vascular dementia, stroke or other non-amyloid neurodegenerative disorders. Unlike most previous studies that included MCI patients of all possible causes, the present study specifically focused on MCI patients with an AD pathology, e.g., amyloid deposition on PET, who we referred to as patients with MCI due to AD. In particular, the precuneus and PCC, which constitute central components of the default mode network (DMN), predominantly showed hypoperfusion in patients with MCI due to AD compared with patients with MCI due to non-AD conditions in this study. The precuneus and PCC are one of the most interconnected hub regions in the human brain and fosters efficient communication with the medial temporal lobe (MTL) network (e.g., hippocampus), strongly contributing to memory processes and executive function that are usually impaired in early stages of AD [34]. Therefore, the PCC and adjacent medial precuneus exhibited a high dependence on aerobic glycolysis and CBF [35] during both resting and cognitive task-related states, making these areas prone to ischemia and vulnerable to early AD pathology [36].

The relatively decreased CBF in MCI patients might reflect vascular dysfunction and neuronal degeneration in the early stages of AD [37,38]. A reduction in CBF might be an early event in AD, stemming from the constriction of capillaries by contractile pericytes, which are likely evoked by oligomeric Aβ [39]. A data-driven analysis of images and fluid biomarkers indicated that CBF changes assessed with ASL MRI occurred even before Aβ and tau changes emerged in cognitively unimpaired and impaired individuals [40]; this finding was also supported by studies of transgenic mice that found that capillary dysfunction preceded Aβ deposition and memory impairment in the APP/PS1 mice of different ages [41]. In addition, several epidemiological studies observed that vascular factors accelerating cerebrovascular injury, such as hypertension, diabetes mellitus, dyslipidemia, smoking, and obesity, not only directly lead to vascular cognitive impairment but also contribute to neurodegenerative dementias, in particular AD [42]. Furthermore, vascular dysfunction (e.g., hypoxia and ischemia resulting from cerebral hypoperfusion) could lead to increases in not only Aβ and tau deposition but also neuroinflammatory responses and neuronal and glial cell apoptosis, contributing to the development of neurodegeneration [43].

We also found that relative hyperperfusion mainly occurred in the primary cortex and deep GM in patients with MCI due to AD after adjusting for the global CBF value. Previous studies have also reported relatively increased CBF in certain brain regions, including some frontal and temporal areas [18], the hippocampus, and deep GM, e.g., the lentiform nucleus [22], amygdala, and caudate nucleus [20], in patients clinically diagnosed with MCI, although the details of the results are not completely consistent with ours. Similarly, in addition to global and regional CBF reductions, regions with relatively increased CBF were observed in patients with mild to moderate AD in our previous study, particularly in the primary motor and sensory cortex, frontal supplementary motor area, and bilateral putamen [13]. Such relatively regional hyperperfusion is typically explained as a compensatory mechanism for maintaining neural activity by increasing oxygen and energy levels via the blood supply under pathological conditions [21,44], suggesting that some brain areas are relatively preserved in the early stages of AD. Moreover, topographic hyperperfusion compensatory patterns may vary across the AD continuum [18,45,46]. Increased perfusion in the frontal lobe typically occurs during early cognitive decline [45]; in contrast, other elements of compensatory pathways, such as the basal ganglia, cingulate gyrus, hippocampus, and amygdala, show increased perfusion over the course of cognitive function deterioration [18,47,48]. Interestingly, some regions with relative hyperperfusion in our study (e.g., frontal and lateral temporal areas) have been shown to exhibit an increase in translocator protein 18 kDa (TSPO) uptake on PET images, which is used to assess the activation of microglia, in patients with dementia or MCI due to AD, suggesting that neuroinflammation might be another potential mechanism for hyperperfusion [49]. Taken together, these findings reflect the characteristic perfusion changes, including both a predominant CBF reduction and relative CBF increases in certain regions, that occur at the early stages of AD. The presence of both hypoperfusion and hyperperfusion indicates a redistribution of CBF in MCI patients [50].

Although AD is the most common cause of cognitive impairment and dementia in elderly individuals, a substantial proportion of MCI patients do not have an AD pathology; instead, the MCI is attributable to another pathological condition, such as various types of tauopathy, α-synucleinopathy, cerebrovascular disease, and mental disorders or even normal aging. In our memory clinic and other studies, only approximately 50 % of patients clinically diagnosed with MCI of uncertain etiology had positive results on PET scans [[51], [52], [53], [54]]. In this study, the relative CBF value of the left precuneus exhibited optimal performance in discriminating patients with MCI due to AD from CUCs (AUC=0.80) and from patients with MCI due to non-AD conditions (AUC=0.85). According to these findings, when the relative CBF value of the left precuneus is less than 1.62, MCI is highly suspected to be caused by AD pathology with a specificity of approximately 83 %. The diagnostic ability of cerebral perfusion and metabolism in the region of precuneus/PCC for AD has been investigated in previous studies. A previous study revealed that both glucose metabolism measured with ^18^F-fluorodeoxyglucose (FDG)-PET (AUC=0.71) and the absolute (AUC=0.77) and relative (AUC=0.74) CBF measured with ASL in the PCC showed moderate performance in discriminating patients with clinically diagnosed MCI from older controls [11]. In an early ASL study, the PCC was the only region of interest with different CBF values between patients with clinically diagnosed AD and those with FTD, achieving an AUC of 0.74 in discriminating the two patient groups, whereas the CBF values of the precuneus exhibited the highest diagnostic accuracy (AUC=0.85) in discriminating AD patients from older controls [55]. Moreover, an AD-typical hypometabolic pattern including the temporoparietal region, PCC and precuneus on FDG PET was found to suggest amyloid pathology in patients with cognitive impairment from our memory clinic cohort [56]. Accordingly, our findings based on patients with MCI due to AD support the idea that regional CBF changes in the precuneus extending to the PCC as measured with ASL are a potential biomarker for identifying AD pathology and differentiating these patients from those with a non-AD pathology, even at early stages. Since ASL has been shown to be a promising alternative to the gold standard CBF measurement of [^15^O] H_2_O PET, it is suitable for wider clinical use according to cost-effectiveness and accessibility compared with PET scans; moreover, ASL can be obtained simultaneously with only 5 min of extra scan time when conducting structural MRI, which has become a routine examination for AD in real-world practice.

To our knowledge, this is the first study to validate the utility of the ADRP expression established by SSM/PCA in patients with MCI due to AD. Although the subject ADRP expression score was significantly elevated in patients with MCI due to AD and could discriminate those patients from CUCs, it could not efficiently discriminate MCI patients with and without amyloid deposition. In addition, the diagnostic performance of the ADRP expression score for patients with MCI due to AD was not as effective as that for mild to moderate AD dementia reported in our previous study (AUC=0.67 vs. AUC=0.87) [13]. Since the CBF changes at the MCI stage are not as robust as those at the dementia stage of AD, the perfusion pattern established in AD dementia patients might not be sufficiently sensitive for identifying AD patients at early stages. A perfusion pattern based on patients in the prodromal or preclinical stages may be more useful for early diagnosis and differentiation. Furthermore, Aβ deposition may reach a plateau in the early stages of AD and is not associated with subsequent clinical progression [57]. In contrast, tau pathology has been observed to more closely correlate with AD-related clinical outcomes and contribute to the progression of dementia in AD patients. Aβ-positive individuals with MCI who also exhibited tau deposition on PET scans or elevated levels of phosphorylated tau (p-tau) in the CSF were more likely to progress to dementia compared to those having negative results for tau biomarkers [58]. Therefore, the associations between CBF, ADRP, and tau pathology are worthy of investigation in future studies.

Both ADRP expression and regional CBF showed extensive correlations with various cognitive function scores, particularly those for memory, processing speed, and executive function, which are usually impaired early in AD patients. The ADRP overlapped with some regions of the DMN, such as the precuneus and cingulate cortex, which are vulnerable to early amyloid deposition [36,59] and have been widely shown to be associated with cognitive impairment in AD patients. In our previous study, the ADRP expression score showed stronger and more extensive correlations with various cognitive function scores than did the relative regional CBF value in patients with AD dementia [13]. Since network analysis generally recovers more disease specific and widely distributed brain regions that may not have direct biological correlates than regional analysis does, our findings indicate the potential use of ADRP in monitoring disease progression in patients with symptomatic AD.

## 5 Limitations

This study has several limitations. First, this study was conducted solely in a single medical institution in China with a small sample, facing limitations in generalizability to other populations due to potential cultural, genetic, or environmental (e.g., climate) factors that might impact the outcomes. In addition, although we consecutively recruited MCI participants who first visit our memory clinic and have not received any suggestions for prevention or intervention from dementia specialists before undergoing imaging and cognitive evaluations to mitigate potential selection bias, the included participants may have a stronger awareness of health and more likely have a healthy lifestyle, e.g., regular exercise and a balanced diet, who would not represent real-world populations. Second, the PiB- MCI group had a smaller sample size than the PiB+ group and potentially encompassed diverse underlying causes and pathogeneses, including even normal aging. A larger sample size of PiB- MCI participants is needed to strengthen the conclusions drawn about non-AD-related MCI. In addition, some patients with MCI due to AD, e.g., at very early pathological stages, might present an equivocal or negative manifestation on PiB PET scans, meanwhile, patients with MCI caused by another etiology might have a co-pathology of Aβ and present as PiB-positive on PET scans, leading to misclassifications. Moreover, other possible sources of heterogeneity as potential confounders also exist within the same group, such as lifestyle factors (e.g., diet and physical activity) and APOE genotypes. Furthermore, mixed pathologies with cerebrovascular disease could be also present in our elderly participants, although individuals with moderate or severe stenosis in the large vessels were excluded. Third, although ASL has been suggested to be an alternative to [^15^O] H_2_O PET, CBF maps derived from ASL might not reflect the pure effects of vascular physiology in the brain, especially when blood flow towards the brain is slowed (e.g., a prolonged arterial transit time). Finally, although we observed correlations between perfusion, including both ADRP expression and CBF values, and cognitive function scores, these results were not assessed for repeated measures and reproducibility, and whether perfusion changes predict disease progression in AD remains inconclusive. Therefore, longitudinal studies involving the long-term follow-up of MCI patients would be valuable for validating the clinical significance of ADRP and regional CBF in screening, diagnosing and evaluating early AD.

## 6 Conclusions

In the coming era of disease-modifying therapy, a relatively accessible tool for identifying the AD pathology in the early stages is imperative for improving clinical practice. Our findings suggest that the relative CBF value, particularly in the left precuneus, and the expression of ADRP are promising MRI biomarkers for identifying and monitoring disease progression in early AD patients.

## Declarations

### Ethical approval and consent to participate

The Ethics Committee of Tianjin Medical University General Hospital approved this study. Written informed consent was obtained from all participants.

### Consent for publication

Not applicable.

### Conflict of interest statement

The authors declare no conflicts of interest.

### Data availability

The datasets generated and analyzed during the current study are available from the corresponding author upon reasonable request.

### Funding

This work was supported by Science and Technology Innovation 2030—Major Project (2021ZD0201805) and the Tianjin Key Medical Discipline (Specialty) Construction Project (TJYXZDXK-004A).

### Declaration of generative AI and AI-assisted technologies in the writing process

I hereby confirm that this manuscript was written without the use of any generative AI or AI-assisted technologies. The entire content was crafted solely by the authors, with no assistance from any artificial intelligence tools.

## CRediT authorship contribution statement

**Caixia Wang:** Writing – original draft, Data curation, Investigation, Formal analysis, Validation. **Deli Ji:** Data curation, Investigation. **Xiao Su:** Data curation, Investigation. **Fang Liu:** Formal analysis, Validation. **Yanxin Zhang:** Formal analysis, Validation. **Qingzheng Lu:** Data curation, Investigation. **Li Cai:** Methodology, Project administration. **Ying Wang:** Methodology, Project administration. **Wen Qin:** Methodology, Project administration. **Gebeili Xing:** Formal analysis, Validation. **Peng Liu:** Data curation, Investigation. **Xin Liu:** Data curation, Investigation. **Meili Liu:** Formal analysis, Validation. **Nan Zhang:** Conceptualization, Supervision, Funding acquisition, Methodology, Project administration, Writing – review & editing.

## Declaration of competing interest

The authors declare that they have no competing interests.

## Appendix Supplementary materials

Image, application 1

Image, application 2

Image, application 3

Image, application 4

Image, application 5

## References

[1] (2022). Estimation of the global prevalence of dementia in 2019 and forecasted prevalence in 2050: an analysis for the Global burden of disease study 2019. Lancet Public Health.

[2] Jack C.R., Bennett D.A., Blennow K., Carrillo M.C., Dunn B., Haeberlein S.B. (2018). NIA-AA Research Framework: toward a biological definition of Alzheimer's disease. J. Alzheimer's Ass..

[3] Jack C.R., Andrews J.S., Beach T.G., Buracchio T., Dunn B., Graf A. (2024). Revised criteria for diagnosis and staging of Alzheimer's disease: alzheimer's association workgroup. Alzheimers Dement.

[4] Govindpani K., McNamara L.G., Smith N.R., Vinnakota C., Waldvogel H.J., Faull R.L. (2019). Vascular dysfunction in Alzheimer's disease: a prelude to the pathological process or a consequence of It?. J Clin Med.

[5] Grammas P. (2000). A damaged microcirculation contributes to neuronal cell death in Alzheimer's disease. Neurobiol Aging.

[6] Sweeney M.D., Kisler K., Montagne A., Toga A.W., Zlokovic B.V. (2018). The role of brain vasculature in neurodegenerative disorders. Nat Neurosci.

[7] Montagne A., Nation D.A., Pa J., Sweeney M.D., Toga A.W., Zlokovic B.V (2016). Brain imaging of neurovascular dysfunction in Alzheimer's disease. Acta Neuropathol.

[8] Soldozy S., Galindo J., Snyder H., Ali Y., Norat P., Yağmurlu K. (2019). Clinical utility of arterial spin labeling imaging in disorders of the nervous system. Neurosurg Focus.

[9] Zhang H., Wang Y., Lyu D., Li Y., Li W., Wang Q. (2021). Cerebral blood flow in mild cognitive impairment and Alzheimer's disease: a systematic review and meta-analysis. Ageing Res Rev.

[10] Lou X., Yu S., Scalzo F., Starkman S., Ali L.K., Kim D. (2017). Multi-delay ASL can identify leptomeningeal collateral perfusion in endovascular therapy of ischemic stroke. Oncotarget.

[11] Dolui S., Li Z., Nasrallah I.M., Detre J.A., Wolk D.A. (2020). Arterial spin labeling versus (18)F-FDG-PET to identify mild cognitive impairment. Neuroimage Clin.

[12] Schidlowski M., Stirnberg R., Stöcker T., Rüber T. (2020). Reliability of quantitative transverse relaxation time mapping with [Formula: see text]-prepared whole brain pCASL. Sci Rep.

[13] Meng M., Liu F., Ma Y., Qin W., Guo L., Peng S. (2023). The identification and cognitive correlation of perfusion patterns measured with arterial spin labeling MRI in Alzheimer's disease. Alzheimers Res Ther.

[14] Rafii M.S., Aisen P.S. (2023). Detection and treatment of Alzheimer's disease in its preclinical stage. Nat Aging.

[15] Duan W., Zhou G.D., Balachandrasekaran A., Bhumkar A.B., Boraste P.B., Becker J.T. (2021). Cerebral blood flow predicts conversion of mild cognitive impairment into alzheimer's disease and cognitive decline: an arterial spin labeling follow-up study. J. Alzheimers Dis..

[16] Soman S., Raghavan S., Rajesh P.G., Varma R.P., Mohanan N., Ramachandran S.S. (2021). Relationship between cerebral perfusion on arterial spin labeling (ASL) MRI with brain volumetry and cognitive performance in mild cognitive impairment and dementia due to Alzheimer's disease. Ann Indian Acad Neurol.

[17] Marterstock D.C., Knott M.F.X., Hoelter P., Lang S., Oberstein T., Kornhuber J. (2022). Pulsed arterial spin labeling and segmented brain volumetry in the diagnostic evaluation of frontotemporal dementia, Alzheimer's disease and mild cognitive impairment. Tomography (Ann Arbor, Mich).

[18] Ding B., Ling H.W., Zhang Y., Huang J., Zhang H., Wang T. (2014). Pattern of cerebral hyperperfusion in Alzheimer's disease and amnestic mild cognitive impairment using voxel-based analysis of 3D arterial spin-labeling imaging: initial experience. Clin Interv Aging.

[19] Johnson N.A., Jahng G.H., Weiner M.W., Miller B.L., Chui H.C., Jagust W.J. (2005). Pattern of cerebral hypoperfusion in Alzheimer disease and mild cognitive impairment measured with arterial spin-labeling MR imaging: initial experience. Radiology.

[20] Dai W., Lopez O.L., Carmichael O.T., Becker J.T., Kuller L.H., Gach H.M. (2009). Mild cognitive impairment and alzheimer disease: patterns of altered cerebral blood flow at MR imaging. Radiology.

[21] Wang X., Ding D., Zhao Q., Liang X., Peng L., Zhao X. (2020). Brain hemodynamic changes in amnestic mild cognitive impairment measured by pulsed arterial spin labeling. Aging.

[22] Tang T., Huang L., Zhang Y., Li Z., Liang S. (2022). Aberrant pattern of regional cerebral blood flow in mild cognitive impairment: a meta-analysis of arterial spin labeling magnetic resonance imaging. Front Aging Neurosci.

[23] Roquet D., Sourty M., Botzung A., Armspach J.P., Blanc F. (2016). Brain perfusion in dementia with Lewy bodies and Alzheimer's disease: an arterial spin labeling MRI study on prodromal and mild dementia stages. Alzheimers Res Ther.

[24] Sachdev P.S., Blacker D., Blazer D.G., Ganguli M., Jeste D.V., Paulsen J.S. (2014). Classifying neurocognitive disorders: the DSM-5 approach. Nat Rev Neurol.

[25] Albert M.S., DeKosky S.T., Dickson D., Dubois B., Feldman H.H., Fox N.C. (2011). The diagnosis of mild cognitive impairment due to Alzheimer's disease: recommendations from the National Institute on Aging-Alzheimer's Association workgroups on diagnostic guidelines for Alzheimer's disease. Alzheimers Dement.

[26] Jian W., Yi-Zhuang Z., Jie-Feng C., Hong-Zhen F., Nan C., Jing Y., et al. Reliability and construct validity of the Chinese version of the Wechsler Adult Intelligence Scale-Fourth Edition. 2013.

[27] Heinrich J., Vidal J.S., Simon A., Rigaud A.S., Hanon O., Epelbaum J. (2018). Relationships between lower olfaction and brain white matter lesions in elderly subjects with mild cognitive impairment. J. Alzheimers Dis..

[28] Tian R., Zhang Y., Liu F., Xue X., Zhang Y., Tian Z. (2023). A neuropsychological profile and its correlation with neuroimaging markers in patients with subcortical ischaemic vascular dementia. Int J Geriatr Psychiatry.

[29] Wang Y., Shi Z., Zhang N., Cai L., Li Y., Yang H. (2019). Spatial patterns of hypometabolism and amyloid deposition in variants of alzheimer's disease corresponding to brain networks: a prospective cohort study. Mol Imaging Biol.

[30] Zhang N., Zhang L., Li Y., Gordon M.L., Cai L., Wang Y. (2017). Urine AD7c-NTP predicts amyloid deposition and symptom of agitation in patients with alzheimer's disease and mild cognitive impairment. J. Alzheimers Dis..

[31] Spetsieris P., Ma Y., Peng S., Ko J.H., Dhawan V., Tang C.C. (2013). Identification of disease-related spatial covariance patterns using neuroimaging data. J Vis Exp.

[32] Sierra-Marcos A. (2017). Regional cerebral blood flow in mild cognitive impairment and Alzheimer's disease measured with arterial spin labeling magnetic resonance imaging. Int J Alzheimers Dis.

[33] Zhang N., Gordon M.L., Goldberg T.E. (2017). Cerebral blood flow measured by arterial spin labeling MRI at resting state in normal aging and Alzheimer's disease. Neurosci Biobehav Rev.

[34] Chirles T.J., Reiter K., Weiss L.R., Alfini A.J., Nielson K.A., Smith J.C. (2017). Exercise training and functional connectivity changes in mild cognitive impairment and healthy elders. J. Alzheimers Dis..

[35] Vlassenko A.G., Vaishnavi S.N., Couture L., Sacco D., Shannon B.J., Mach R.H. (2010). Spatial correlation between brain aerobic glycolysis and amyloid-β (Aβ) deposition. Proc. Natl. Acad. Sci. U.S.A..

[36] Ali D.G., Bahrani A.A., Barber J.M., El Khouli R.H., Gold B.T., Harp J.P. (2022). Amyloid-PET levels in the precuneus and posterior cingulate cortices are associated with executive function scores in preclinical Alzheimer's disease prior to overt global amyloid positivity. J. Alzheimers Dis..

[37] Popa-Wagner A., Buga A.M., Popescu B., Muresanu D. (2015). Vascular cognitive impairment, dementia, aging and energy demand. A vicious cycle. J Neural Transm (Vienna).

[38] Kisler K., Nelson A.R., Montagne A., Zlokovic B.V. (2017). Cerebral blood flow regulation and neurovascular dysfunction in Alzheimer disease. Nat Rev Neurosci.

[39] Korte N., Nortley R., Attwell D. (2020). Cerebral blood flow decrease as an early pathological mechanism in Alzheimer's disease. Acta Neuropathol.

[40] Iturria-Medina Y., Sotero R.C., Toussaint P.J., Mateos-Pérez J.M., Evans A.C., Weiner M.W. (2016). Early role of vascular dysregulation on late-onset Alzheimer's disease based on multifactorial data-driven analysis. Nat Commun.

[41] Wang N.Y., Li J.N., Liu W.L., Huang Q., Li W.X., Tan Y.H. (2021). Ferulic acid ameliorates alzheimer's disease-like pathology and repairs cognitive decline by preventing capillary hypofunction in APP/PS1 mice. Neurotherapeutics.

[42] Pasqualetti G., Thayanandan T., Edison P. (2022). Influence of genetic and cardiometabolic risk factors in Alzheimer's disease. Ageing Res Rev.

[43] Apátiga-Pérez R., Soto-Rojas L.O., Campa-Córdoba B.B., Luna-Viramontes N.I., Cuevas E., Villanueva-Fierro I. (2022). Neurovascular dysfunction and vascular amyloid accumulation as early events in Alzheimer's disease. Metab Brain Dis.

[44] Howarth C. The contribution of astrocytes to the regulation of cerebral blood flow. 2014;8. 10.3389/fnins.2014.00103. PMC4023041 24847203

[45] Clément F., Belleville S. (2010). Compensation and disease severity on the memory-related activations in mild cognitive impairment. Biol Psychiatry.

[46] Park D.C., Reuter-Lorenz P. (2009). The adaptive brain: aging and neurocognitive scaffolding. Annu Rev Psychol.

[47] Alsop D.C., Casement M., de Bazelaire C., Fong T. (2008). Press DZ. Hippocampal hyperperfusion in Alzheimer's disease. Neuroimage.

[48] Chen W., Song X., Beyea S., D'Arcy R., Zhang Y., Rockwood K (2011). Advances in perfusion magnetic resonance imaging in Alzheimer's disease. Alzheimers Dement.

[49] Leng F., Hinz R., Gentleman S., Hampshire A., Dani M., Brooks D.J. (2023). Neuroinflammation is independently associated with brain network dysfunction in Alzheimer's disease. Mol Psychiatry.

[50] Camargo A., Wang Z. (2023). Hypo- and hyper-perfusion in MCI and AD identified by different ASL MRI sequences. Brain Imaging Behav.

[51] Wang Y., Lou F., Li Y., Liu F., Wang Y., Cai L. (2021). Clinical, neuropsychological, and neuroimaging characteristics of amyloid- positive vs. Amyloid-negative patients with clinically diagnosed alzheimer's disease and amnestic mild cognitive impairment. Curr Alzheimer Res.

[52] Rabinovici G.D., Gatsonis C., Apgar C., Chaudhary K., Gareen I., Hanna L. (2019). Association of amyloid positron emission tomography with subsequent change in clinical management among medicare beneficiaries with mild cognitive impairment or dementia. JAMA.

[53] Collij L.E., Salvadó G., de Wilde A., Altomare D., Shekari M., Gispert J.D. (2023). Quantification of [(18) F]florbetaben amyloid-PET imaging in a mixed memory clinic population: the ABIDE project. Alzheimers Dement.

[54] Leuzy A., Savitcheva I., Chiotis K., Lilja J., Andersen P., Bogdanovic N. (2019). Clinical impact of [(18)F]flutemetamol PET among memory clinic patients with an unclear diagnosis. Eur J Nucl Med Mol Imaging.

[55] Steketee R.M., Bron E.E., Meijboom R., Houston G.C., Klein S., Mutsaerts H.J. (2016). Early-stage differentiation between presenile Alzheimer's disease and frontotemporal dementia using arterial spin labeling MRI. Eur Radiol.

[56] Liu F., Shi Y., Wu Q., Chen H., Wang Y., Cai L. (2024). The value of FDG combined with PiB PET in the diagnosis of patients with cognitive impairment in a memory clinic. CNS Neurosci Ther.

[57] Brosseron F., Krauthausen M., Kummer M., Heneka M.T. (2014). Body fluid cytokine levels in mild cognitive impairment and Alzheimer's disease: a comparative overview. Mol Neurobiol.

[58] Huszár Z., Engh M.A., Pavlekovics M., Sato T., Steenkamp Y., Hanseeuw B. (2024). Risk of conversion to mild cognitive impairment or dementia among subjects with amyloid and tau pathology: a systematic review and meta-analysis. Alzheimers Res Ther.

[59] Miners J.S., Palmer J.C., Love S. (2016). Pathophysiology of hypoperfusion of the precuneus in early Alzheimer's disease. Brain Pathol.

