# Supplementary material for: Cerebral perfusion correlates with amyloid deposition in patients with mild cognitive impairment due to Alzheimer's disease
Source: J Prev Alzheimers Dis. 2025 Jan 1;12(2):100031. doi: 10.1016/j.tjpad.2024.100031 (PMC12183967; doi:10.1016/j.tjpad.2024.100031)
Supplement: Supplementary file 1 [file mmc1.docx]

**Supplementary Table 1 Demographics and cognitive performance of all participants**

|  | MCI  N=59 | CUC  N=49 | F/χ2 | P |  |
| --- | --- | --- | --- | --- | --- |
| Age, years | | 68.81 (5.72) | 67.45 (5.51) | F=1.08 | 0.21 |
| Sex, F/M | | 47/12 | 29/20 | χ2=4.45 | 0.04 |
| Education, years | | 12.19(3.04) | 11.82 (3.53) | F=1.34 | 0.61 |
| Handedness, L/R | | 3/56 | 1/48 | χ2=0.10 | 0.75 |
| Hypertension | | 22/37 | 21/28 | χ2=0.15 | 0.70 |
| Hyperlipidaemia | | 12/47 | 9/40 | χ2=0.66 | 0.80 |
| Obesity | | 2/57 | 5/44 | χ2=1.08 | 0.30 |
| Smoking | | 2/57 | 2/47 | χ2=0.36 | 0.85 |

MCI, mild cognitive impairment; CUC, cognitively unimpaired control.
