# Supplementary material for: Cerebral perfusion correlates with amyloid deposition in patients with mild cognitive impairment due to Alzheimer's disease
Source: J Prev Alzheimers Dis. 2025 Jan 1;12(2):100031. doi: 10.1016/j.tjpad.2024.100031 (PMC12183967; doi:10.1016/j.tjpad.2024.100031)
Supplement: Supplementary file 2 [file mmc2.docx]

**Supplementary Table 2** Main regions showing decreased CBF in patients with MCI due to AD with respect to CUCs (without normalization)

| Structure | BA | X | Y | Z | T | Cluster size (ml) |
| --- | --- | --- | --- | --- | --- | --- |
| Left fusiform | 37 | -24 | -9 | 36 | 4.04 | 1.69 |
| Left inferior parietal lobule | 40 | -44 | -50 | 53 | 3.58 | 0.72 |
| Left middle cingulate cortex | 23 | -2 | -36 | 35 | 3.43 | 0.91 |
| Right angular gyrus | 39 | 42 | -57 | 39 | 3.35 | 0.31 |

Significant clusters were defined as those for which the uncorrected *P* < 0.001.

CBF, cerebral blood flow; MCI, mild cognitive impairment; AD, Alzheimer’s disease; CUC, cognitively unimpaired control; BA, Brodmann area.
