# Supplementary material for: Cerebral perfusion correlates with amyloid deposition in patients with mild cognitive impairment due to Alzheimer's disease
Source: J Prev Alzheimers Dis. 2025 Jan 1;12(2):100031. doi: 10.1016/j.tjpad.2024.100031 (PMC12183967; doi:10.1016/j.tjpad.2024.100031)
Supplement: Supplementary file 3 [file mmc3.docx]

**
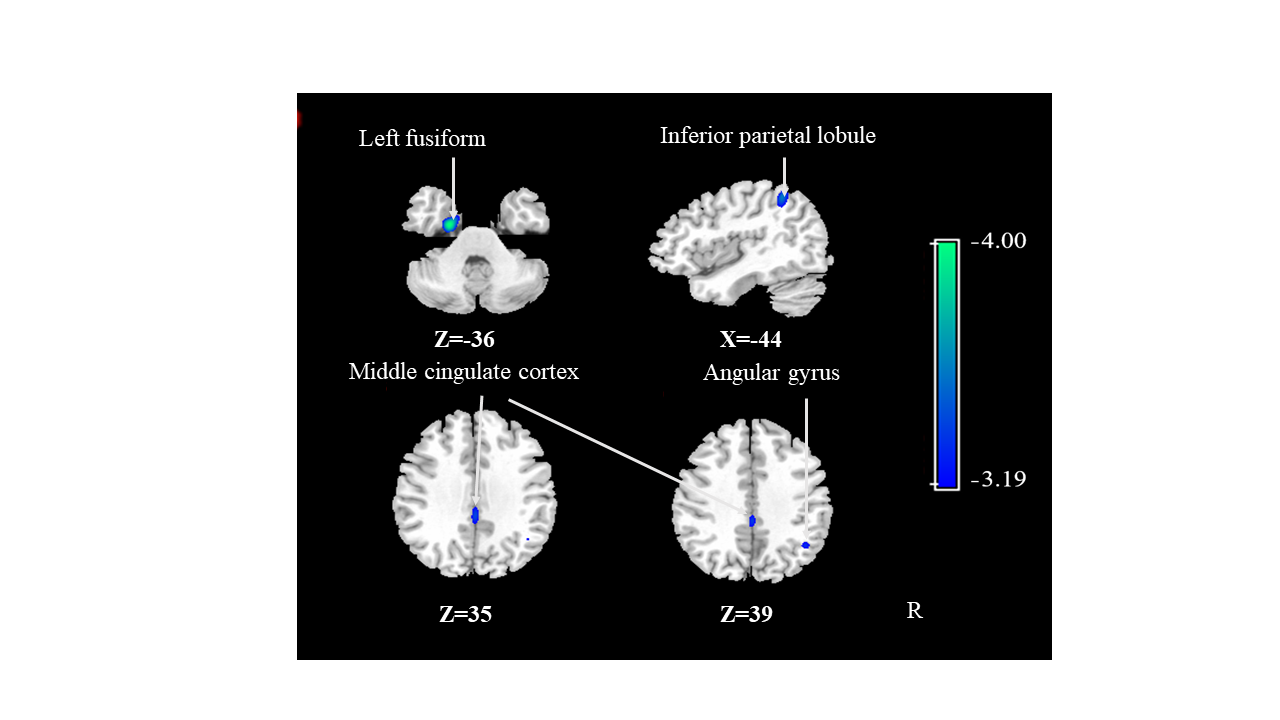
Supplementary Fig. 1** Regional changes in CBF from univariate analysis prior to adjusting for the global value in patients with MCI due to AD compared with CUCs. Cold colors indicate regions with decreased CBF in patients with MCI due to AD with respect to CUCs. A threshold of 3.19 (*P* < 0.001, uncorrected) was used to overlay the SPM maps onto a standard MRI brain template. CBF, cerebral blood flow; MCI, mild cognitive impairment; AD, Alzheimer’s disease; CUC, cognitively unimpaired control; SPM, statistical parametric mapping; MRI, magnetic resonance angiography.
