# Supplementary material for: Cerebral perfusion correlates with amyloid deposition in patients with mild cognitive impairment due to Alzheimer's disease
Source: J Prev Alzheimers Dis. 2025 Jan 1;12(2):100031. doi: 10.1016/j.tjpad.2024.100031 (PMC12183967; doi:10.1016/j.tjpad.2024.100031)
Supplement: Supplementary file 4 [file mmc4.docx]

**
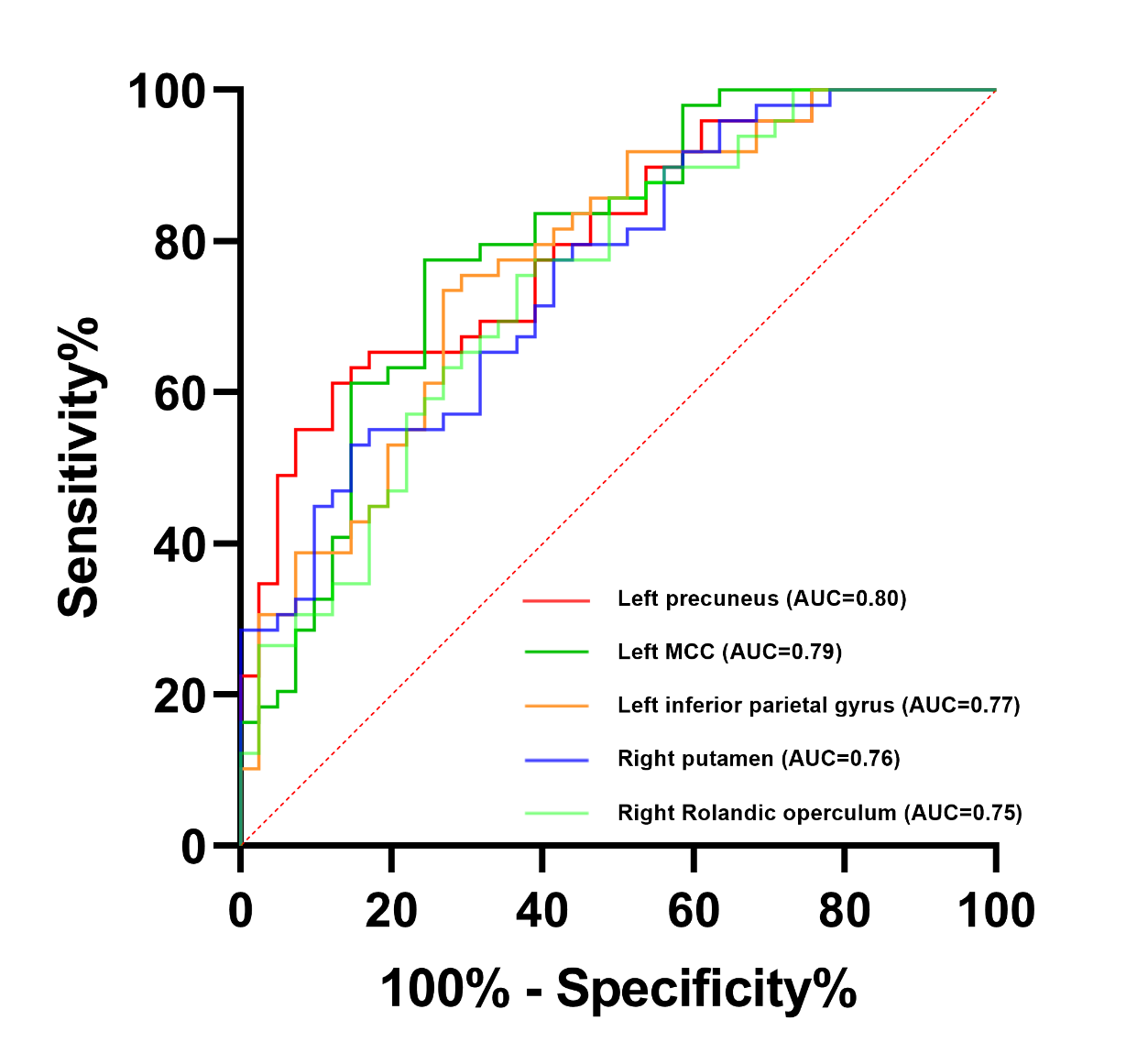
**

**Supplementary Fig. 2** ROC curves of relative CBF values in distinguishing patients with MCI due to AD from CUCs. Representative regions with AUCs > 0.75 are shown. ROC, receiver operating characteristic; CBF, cerebral blood flow; MCI, mild cognitive impairment; AD, Alzheimer’s disease; CUC, cognitively unimpaired control; AUC, area under the curve; MCC, middle cingulate cortex.
