# Supplementary material for: Cerebral perfusion correlates with amyloid deposition in patients with mild cognitive impairment due to Alzheimer's disease
Source: J Prev Alzheimers Dis. 2025 Jan 1;12(2):100031. doi: 10.1016/j.tjpad.2024.100031 (PMC12183967; doi:10.1016/j.tjpad.2024.100031)
Supplement: Supplementary file 5 [file mmc5.docx]

**
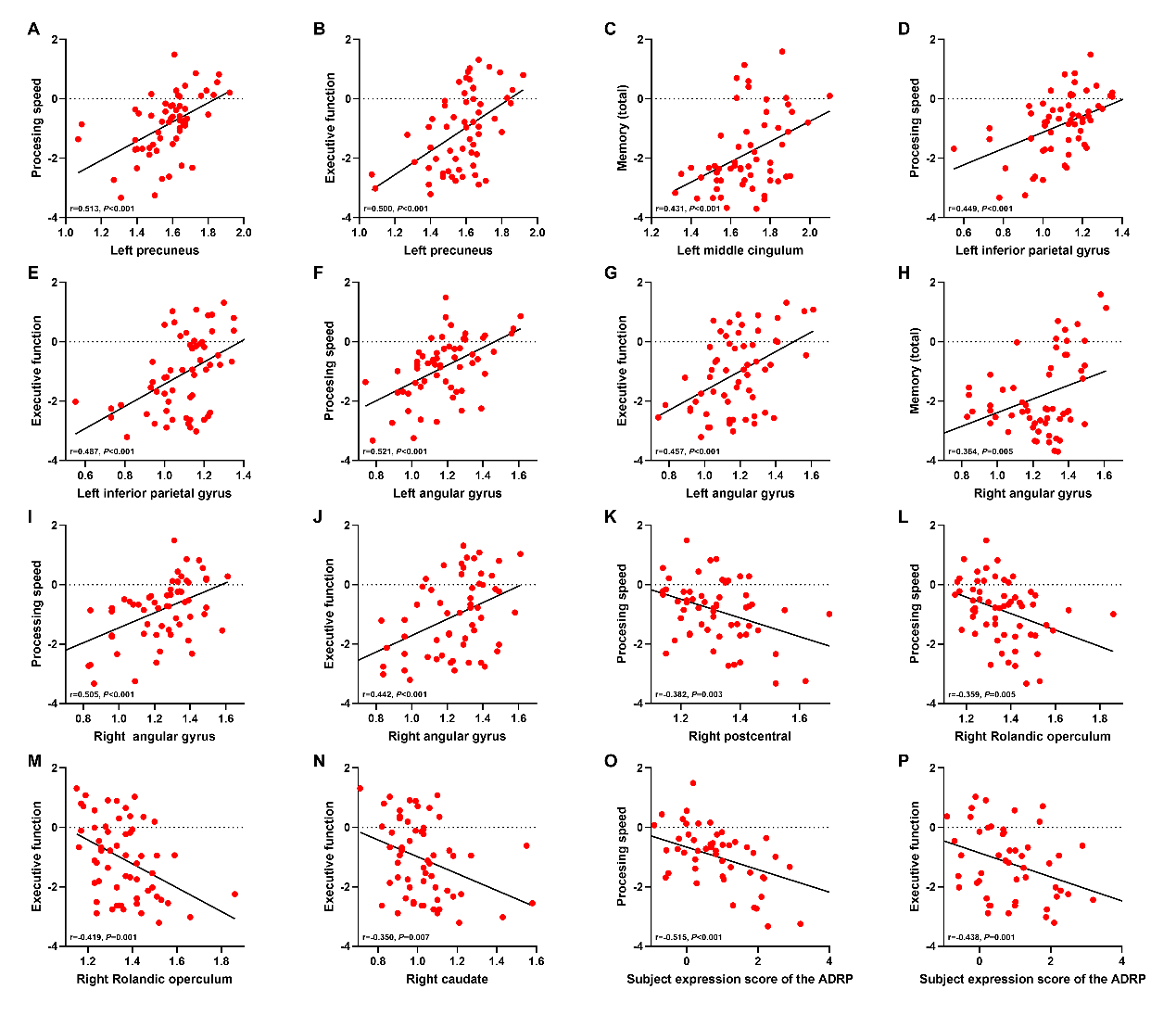
**

**Supplementary Fig. 3** Correlations between relative regional CBF, the subject expression score of the ADRP and cognitive function scores in all patients with MCI. The raw scores were converted to z scores for the subject expression scores of the ADRP and the scores of different cognitive domains. Pearson correlation analysis with subsequent Bonferroni correction for multiple comparisons was conducted. CBF, cerebral blood flow; ADRP, AD-related perfusion pattern; MCI, mild cognitive impairment.
